# Cancer suspicion in general practice, urgent referral and time to diagnosis: a population-based GP survey and registry study

**DOI:** 10.1186/1471-2407-14-636

**Published:** 2014-08-30

**Authors:** Henry Jensen, Marie Louise Tørring, Frede Olesen, Jens Overgaard, Peter Vedsted

**Affiliations:** Research Unit for General Practice, Research Centre for Cancer Diagnosis in Primary Care, Department of Public Health, Aarhus University, Bartholins Allé 2, DK-8000 Aarhus C, Denmark; Section for General Medical Practice, Department of Public Health, Aarhus University, Bartholins Allé 2, DK-8000 Aarhus C, Denmark; Department of Experimental Clinical Oncology, Aarhus University Hospital, Noerrebrogade, DK-8000 Aarhus C, Denmark

**Keywords:** Fast-track, Neoplasm, (Early) diagnosis, General practice, Delay, Cancer suspicion, Denmark

## Abstract

**Background:**

Many countries have implemented standardised cancer patient pathways (CPPs) to ensure fast diagnosis of patients suspected of having cancer. Yet, studies are sparse on the impact of such CPPs, and few have distinguished between referral routes. For incident cancer patients, we aimed to determine how often GPs suspected cancer at the time of first presentation of symptoms in general practice and to describe the routes of referral for further investigation. In addition, we aimed to analyse if the GP’s suspicion of cancer could predict the choice of referral to a CPP. Finally, we aimed to analyse associations between not only cancer suspicion and time to cancer diagnosis, but also between choice of referral route and time to cancer diagnosis.

**Methods:**

We conducted a population-based, cross-sectional study of incident cancer patients in Denmark who had attended general practice prior to their diagnosis of cancer. Data were collected from GP questionnaires and national registers. We estimated the patients’ chance of being referred to a CPP (prevalence ratio (PR)) using Poisson regression. Associations between the GP’s symptom interpretation, use of CPP and time to diagnosis were estimated using quantile regression.

**Results:**

5,581 questionnaires were returned (response rate: 73.8%). A GP was involved in diagnosing the cancer in 4,101 (73.5%) cases (3,823 cases analysed). In 48.2% of these cases, the GP interpreted the patient’s symptoms as ‘alarm’ symptoms suggestive of cancer. The GP used CPPs in 1,426 (37.3%) cases. Patients, who had symptoms interpreted as ‘vague’ had a lower chance of being referred to a CPP than when interpreted as ‘alarm’ symptoms (PR = 0.53 (95%CI: 0.48;0.60)). Patients with ‘vague’ symptoms had a 34 (95% CI: 28;41) days longer median time to diagnosis than patients with ‘alarm’ symptoms.

**Conclusions:**

GPs suspect cancer more often than they initiate a CPP, and patients were less likely to be referred to a CPP when their symptoms were not interpreted as alarm symptoms of cancer. The GP’s choice of referral route was a strong predictor of the duration of the diagnostic interval, but the GP’s symptom interpretation was approximately twice as strong an indicator of a longer diagnostic interval.

## Background

Standardised cancer patient pathways (CPPs) have been implemented during the last decade in many countries, including Denmark, to ensure fast diagnosis of patients suspected to have cancer. This strategy is intended to improve patient satisfaction, reduce waiting times and ensure earlier and faster diagnosis, which should ultimately improve the patient’s prognosis [1–7]. Even though the contents of the CPPs differ between countries, all CPPs operate with criteria-based suspicion of cancer and a guaranteed timeframe.

The UK have introduced two-week wait referrals (2WW): referrals where the GP suspects cancer and refers the patient as urgent, meaning the patient should be seen by a specialist within two weeks. To qualify to be referred as urgent to a 2WW, the patient need to fulfil the criteria outlined in the NICE guidelines. Previous studies of the British 2WW referrals have shown that the general practitioners’ (GPs) use of these referrals was from one in five to one in three of cancer patients and that patients not referred urgently had significantly longer duration of the time to diagnosis [8–13].

In 2007–2009, CPPs were introduced in Denmark for diagnosis and treatment of suspected cancer as part of the Danish National Cancer Plan II [2, 14]. The Danish CPPs consisted of guidelines, descriptions of selected alarm symptoms that may raise cancer suspicion and well-defined diagnosing schedules from clinical suspicion of cancer until treatment, including specific time frames; hence the Danish CPPs can be seen as comparable to the 2WW in the UK. The five Danish regions (i.e. the hospital owners) were given three months to implement the guidelines at local level [2]. By spring 2009, CPPs for 32 specific cancers had been developed [2, 3].

A key issue for assessment of CPPs is knowledge about the decisions behind the timing of CPP initiation for a particular patient. Danish GPs can refer patients to a CPP when a so-called ‘reasonable suspicion of cancer’ is raised. This suspicion rests on a combination of evidence and consensus regarding the possibility of having cancer when presenting a specific alarm symptom of cancer in combination with preliminary test results for certain age groups [2]. Traditionally, ‘alarm’ symptoms and signs of cancer have been derived from cancer patients symptomatology when the diagnosis has been established, but many symptoms of cancer are both benign and highly prevalent in the general population and are often presented in general practice [15, 16]. This may raise concerns as to whether the GP is able to raise a suspicion of cancer based upon the patient’s symptoms. Furthermore, it is unknown if the GP may decide to refer to fast-track diagnosis without ‘alarm’ symptoms or not. Most previous studies have focused solely on cancer patients with at least one recorded alarm symptom of cancer [17, 18] even though many cancer patients do not present alarm symptoms [19, 20]. Consequently, we need more knowledge on how GPs interpret the symptomatology of the full range of cancer patients and who the GP choses to refer to a CPP. In addition, we need to know more about the GP’s handling of cancer suspicion and how this may influence the time to diagnosis.

For these reasons we hypothesized, that when the GPs’ suspected cancer based upon the patient’s symptoms the GP would be more likely to use a CPP than when the GP did not suspect cancer. Furthermore we suspected that this would influence the duration of the diagnostic interval by longer diagnostic intervals for those patients, where the GP did not suspect cancer and also for those patients not referred to a CPP.

For incident cancer patients, we aimed to determine how often GPs suspected cancer at the time of first presentation of symptoms in general practice and to describe the routes of referral for further investigation. In addition, we aimed to analyse if the GP’s suspicion of cancer could predict the choice of referral to a CPP. Finally, we aimed to analyse associations between not only cancer suspicion and time to cancer diagnosis, but also between choice of referral route and time to cancer diagnosis.

## Methods

We conducted a population-based cross-sectional study of incident cancer patients who attended Danish general practice prior to the cancer diagnosis.

### Setting

Denmark has a population of approximately 5.6 million people and an annual cancer incidence rate of 326 per 100,000 [21]. All citizens in Denmark have free access to diagnosis and treatment services through the publicly funded health-care system. Around 98% of all Danish citizens are listed with a general practice [22], and GPs initiate diagnostics and act as gatekeepers to specialized medical care. Danish GPs are legally bound to keep detailed and contemporaneously updated electronic medical records of their patients.

### Study population

**Figure 1:**
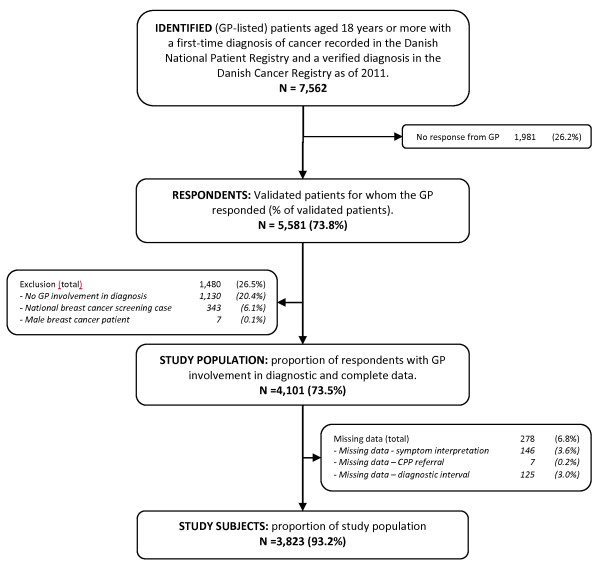
**Flowchart showing patient inclusion.** Boxes on the left indicate exclusion of patients, while boxes on the right indicate drop-outs.

We identified all patients aged 18 years or more with an incident diagnosis of cancer, except for non-melanoma skin cancer, during four months (1 May to 31 August 2010). The study population was subsequently restricted to the 73.5% of patients who, according to the GP, had attended general practice as part of the cancer diagnosis (Figure 1). The remaining patients were diagnosed through screening (6.1%), emergency access or as coincidental findings during diagnostics of other illnesses.

### Identification of patients

Patients were identified using a validated algorithm [23] that uses data from the Danish National Patient Register (NPR) of all inpatient and outpatient visits and diagnoses defined in accordance with the 10th version of the International Classification of Diseases (ICD-10) [24]. We verified all diagnoses by linking data to the Danish Cancer Registry (DCR) [25]. An incident cancer was defined as having a cancer diagnosis as the primary diagnosis (except for non-melanoma skin cancer) and no prior history of cancer recorded in the DCR (previous non-melanoma skin cancer was allowed).

### Data collection

We collected data for each patient by mailing a questionnaire to the patient’s GP, who was asked to fill out the questionnaire on the basis of the medical records. The participating GPs received no remuneration. Non-responders received a reminder, including a new questionnaire, after five weeks.

The questionnaire focused on information about the GP’s interpretation of the symptoms presented by the patient at the first consultation by asking the GP: ‘How did you interpret the symptoms?’ The GP was given three possible categories to answer: alarm symptoms suggestive of cancer (alarm), symptoms suggestive of any serious disease (serious), or vague symptoms not directly suggestive of cancer or other serious disease (vague). Thus, the category of alarm symptoms mirrors the GP’s suspicion of cancer. However, the GP’s symptom interpretation was subjective and was not based on a pre-specified list of alarm symptoms.

The questionnaire also requested information about the choice of referral for further investigation for cancer, i.e. whether or not a referral was made to a CPP. If no referral to a CPP had been made, the questionnaire focused on information about the patient’s referral to specialist care. This enabled us to classify the GP’s choice of referral into the following four distinct categories: Cancer Patient Pathway (CPP), cancer obs. pro. but no cancer patient pathway, other, or unknown referral.

We defined the diagnostic interval as the time interval from the date of the patient’s first presentation of symptoms in primary care until the date of diagnosis in accordance with the Aarhus Statement [26]. The date of the patient’s first presentation of symptoms in primary care was identified by asking the GP the following question: *“When did the patient first present to your practice with symptom(s) that you thought were related to the current cancer diagnosis? (date)”* [26]. The date of diagnosis was obtained from the DCR; this date corresponds to the date of the first contact (admission date) with the hospital department where the cancer diagnosis was first registered as the primary cause of contact or, if the patient was diagnosed by a private practicing specialist, this date corresponds to the date of the clinical diagnosis [27]. If the date of diagnosis was missing in the DCR, the admission date from the NPR was used.

Possible confounders considered were gender, age, comorbidity, educational level, disposable income and region of residence. Patient gender and age were derived from the patient’s civil registry number (CRN), while citizenship was derived from the Danish Civil Registration System [28]. The patient’s complete hospital discharge history (from ten years before the date of the first consultation with the GP) was used to compute a modified Charlson Comorbidity Index (CCI) score in accordance with Quan et al. [29]. We grouped CCI into ‘none’ (no recorded disease), ‘moderate’ (index scores of 1 and 2) and ‘high’ (index scores of 3 or more). We used information on education from Statistics Denmark [30] to identify the educational level of all patients in accordance with the International Standard Classification of Education (ISCED) [31]. We grouped levels of education into ‘low’ (ISCED levels 1 and 2), ‘medium’ (ISCED levels 3 and 4) and ‘high’ (ISCED levels 5 and 6). Finally, the disposable OECD household income level [32] was divided into three categories (‘low’, ‘medium’ and ‘high’) on the basis of data from Statistics Denmark.

More detailed information of identification of patients, data collection and data items are described elsewhere [33].

### Ethical approval

The study was approved by the Danish Data Protection Agency (rec. no. 2009-41-3471). The Danish National Board of Health (today the Danish Health and Medicines Authority) gave, according to section 46 of the Danish Health Act, legal permission to obtain information from the GPs’ medical records, by questionnaires, without the patients’ consent (rec. no. 7-604-04-2/195/EHE). According to Danish law and the Central Denmark Region Committees on Health Research Ethics, approval by the National Committee on Health Research Ethics was not required as no biomedical intervention was performed.

### Analyses

We present results for the five most frequent cancers in Denmark (colorectal, lung, malignant melanoma, breast and prostate [34]) and total. Analyses were performed on 3,823 cases with complete data (Figure 1). No imputation of missing data was made. Descriptive analyses were performed using exact non-parametric methods.

We estimated the patients’ likelihood to be referred to a CPP as a function of GP symptom interpretation by calculating the prevalence ratios (PRs) using Poisson regression as we expected the outcome to be frequent [35]. The analyses were adjusted for patient gender, age, co-morbidity, educational level, disposable income and region of residence and for patient clusters at GP level.

We estimated the associations between GP symptom interpretation and diagnostic interval and between use of CPP and diagnostic interval using the ‘qcount’ procedure by Miranda [36] for quantile regression analysis [37] on the smoothed quantiles [38], as we considered the outcome to be count data (discrete). We adjusted for patient gender, age, comorbidity, educational level, disposable income and region of residence. Confidence intervals were calculated using standard errors (SEs) estimated from 1000 repetitions bootstrap.

Statistical significance was set at 0.05 or less, and 95% confidence intervals are shown when appropriate. Analyses were done using Stata® v. 13 (StataCorp LP, College Station, TX, USA).

## Results

We identified 7,562 incident cancer patients who fulfilled the inclusion criteria. A total of 5,581 GP questionnaires were returned (response rate: 73.8%). The response rate was higher for female patients, patients diagnosed with breast cancer and patients with high educational level.

The GPs were not involved in diagnosing the cancer for 1,480 (26.5%) of the cases; 343 (6.1%) of these were detected in connection with the national breast cancer screening programme and 1,130 (20.4%) were detected otherwise. Patients listed with uninvolved GPs were more likely to be women, to be 55–64 years of age, to have higher 1-year survival and to have medium educational level.We excluded 278 (6.8%) patients due to missing information on three main variables: dates (125 patients (3.0%)), use of CPP (7 patients (0.2%)) and GP’s symptom interpretation (146 patients (3.6%)) (Figure 1). The excluded patients were more likely to be diagnosed with prostate cancer or colorectal cancer, to have moderate co-morbidity, to be over 75 years of age and to have distant tumour stage (metastatic cancer).

The analysed patient group thus consisted of 3,823 patients of which 53.3% were males, and 52.5% were 55–74 years of age (Table 1).

**Table 1:** **Characteristics of included patients for whom the GP was involved in the clinical pathway, shown by cancer site and total (N = 3,823)**

	Colorectal	Lung	Malignant melanoma	Breast	Prostate	Other	Total
	n(%)	n(%)	n(%)	n(%)	n(%)	n(%)	n(%)
**All**	612(100)	474(100)	227(100)	518(100)	556(100)	1,436(100)	3,823(100)
**Gender**							
Male	325(53.1)	266(56.1)	100(44.1)	0(0)	556(100)	790(55.0)	2,037(53.3)
Female	287(46.9)	208(43.9)	127(55.9)	518(100)	0(0)	646(45.0)	1,786(46.7)
**Age groups (years):**							
18-44	16(2.6)	3(0.6)	63(27.8)	69(13.3)	0(0)	146(10.2)	297(7.8)
45-54	46(7.5)	42(8.9)	48(21.1)	119(23.0)	20(3.6)	184(12.8)	459(12.0)
55-64	115(18.8)	136(28.7)	43(18.9)	100(19.3)	136(24.5)	325(22.6)	855(22.4)
65-74	219(35.8)	167(35.2)	36(15.9)	91(17.6)	238(42.8)	400(27.9)	1,151(30.1)
> = 75	216(35.3)	126(26.6)	37(16.3)	139(26.8)	162(29.1)	381(26.5)	1,061(27.8)
**GP’s symptom interpretation**							
Alarm	298(48.7)	148(31.2)	121(53.3)	419(80.9)	264(47.5)	592(41.2)	1,842(48.2)
Serious	127(20.8)	162(34.2)	9(4.0)	27(5.2)	59(10.6)	362(25.2)	746(19.5)
Vague	187(30.6)	164(34.6)	97(42.7)	72(13.9)	233(41.9)	482(33.6)	1,235(32.3)
**Referral mode**							
Cancer Patient Pathway (CPP)	222(36.3)	193(40.7)	82(36.1)	324(62.5)	220(39.6)	385(26.8)	1,426(37.3)
Cancer obs – no CPP	108(17.6)	79(16.7)	76(33.5)	92(17.8)	199(35.8)	369(25.7)	923(24.1)
Other	262(42.8)	174(36.7)	61(26.9)	83(16.0)	114(20.5)	613(42.7)	1,307(34.2)
Unknown	20(3.3)	28(5.9)	8(3.5)	19(3.7)	23(4.1)	69(4.8)	167(4.4)
**Co-morbidity** ^**1**^							
None	453(74.0)	323(68.1)	192(84.6)	406(78.4)	422(75.9)	1,088(75.8)	2,884(75.4)
Moderate	132(21.6)	124(26.2)	33(14.5)	93(18.0)	114(20.5)	286(19.9)	782(20.5)
High	27(4.4)	27(5.7)	2(0.9)	19(3.7)	20(3.6)	62(4.3)	157(4.1)
Missing	0(0)	0(0)	0(0)	0(0)	0(0)	0(0)	0(0)
**Educational level – ISCED** ^**2**^							
Low	237(38.7)	211(44.5)	61(26.9)	179(34.6)	182(32.7)	551(38.4)	1,421(37.2)
Medium	230(37.6)	170(35.9)	93(41.0)	202(39.0)	236(42.4)	558(38.9)	1,489(38.9)
High	112(18.3)	73(15.4)	67(29.5)	121(23.4)	126(22.7)	263(18.3)	762(19.9)
Missing	33(5.4)	20(4.2)	6(2.6)	16(3.1)	12(2.2)	64(4.5)	151(3.9)
**Disposable income in euro – OECD** ^**3**^							
Low	233(38.1)	198(41.8)	50(22.0)	150(29.0)	138(24.8)	494(34.4)	1,263(33.0)
Medium	209(34.2)	161(34.0)	73(32.2)	184(35.5)	194(34.9)	481(33.5)	1,302(34.1)
High	169(27.6)	115(24.3)	103(45.4)	183(35.3)	224(40.3)	460(32.0)	1,254(32.8)
Missing	1(0.2)	0(0)	1(0.4)	1(0.2)	0(0)	1(0.1)	4(0.1)
**Region of residence** ^**4**^							
North Denmark Region	61(10.0)	43(9.1)	24(10.6)	60(11.6)	81(14.6)	178(12.4)	447(11.7)
Central Denmark Region	141(23.0)	120(25.3)	48(21.1)	129(24.9)	148(26.6)	343(23.9)	929(24.3)
Region of Southern Denmark	139(22.7)	101(21.3)	86(37.9)	128(24.7)	112(20.1)	321(22.4)	887(23.2)
Capital Region of Denmark	142(23.2)	128(27.0)	53(23.3)	109(21.0)	130(23.4)	359(25.0)	921(24.1)
Region Zealand	129(21.1)	82(17.3)	16(7.0)	92(17.8)	85(15.3)	235(16.4)	639(16.7)

^1^Charlson’s Comorbidity index, ^2^ISCED = International Standard Classification of Education, ^3^Disposable income (in thousand of euro according to OECD classification, ^4^Region of the patient’s GP as of November 2010.

### Cancer suspicion and use of CPPs

In 48.2% of the cases, the GP interpreted the patient’s symptom as an ‘alarm’ symptom. This ranged from 31.2% for lung cancer patients to 80.9% for breast cancer patients (Table 1). The GP used CPPs in 1,426 (37.3%) of all cases, ranging from 36.1% for malignant melanoma patients to 62.5% for breast cancer patients (Table 1). The GP used CPPs in 52.0% of the cases, who had symptoms interpreted to be ‘alarm’ symptoms with variation among the different cancer sites (Table 2).

**Table 2:** **Number and percentages of Cancer Patient Pathways (CPP) used among patients for whom the GP was involved in the diagnosis, shown by cancer site and total (N = 1,426)**

	Colorectal	Lung	Malignant melanoma	Breast	Prostate	Other	Total
	n(%)	n(%)	n(%)	n(%)	n(%)	n(%)	n(%)
**Total**	222(36.3)	193(40.7)	82(36.1)	324(62.5)	220(39.6)	385(26.8)	1,426(37.3)
**Gender**							
Male	130(40.0)	110(41.4)	38(38.0)	0(0)	220(39.6)	230(29.1)	728(35.7)
Female	92(32.1)	83(39.9)	44(34.6)	324(62.5)	0(0)	155(24.0)	698(39.1)
**Age groups(years):**							
18-44	6(37.5)	1(33.3)	22(34.9)	37(53.6)	0(0)	39(26.7)	105(35.4)
45-54	12(26.1)	13(31.0)	15(31.3)	67(56.3)	10(50.0)	53(28.8)	170(37.0)
55-64	50(43.5)	58(42.6)	20(46.5)	62(62.0)	58(42.6)	87(26.8)	335(39.2)
65-74	82(37.4)	74(44.3)	14(38.9)	55(60.4)	104(43.7)	102(25.5)	431(37.4)
> = 75	72(33.3)	47(37.3)	11(29.7)	103(74.1)	48(29.6)	104(27.3)	385(36.3)
**GP’s symptom Interpretation**							
Alarm	163(54.7)	74(50.0)	51(42.1)	305(72.8)	121(45.8)	243(41.0)	957(52.0)
Serious	17(13.4)	52(32.1)	0(0)	5(18.5)	20(33.9)	55(15.2)	149(20.0)
Vague	42(22.5)	67(40.9)	31(32.0)	14(19.4)	79(33.9)	87(18.0)	320(25.9)

Referral to a CPP was more likely among male patients than among female patients (PR = 1.12 (95% CI: 1.00-1.24)). Referral to a CPP was less likely among patients, who had symptoms interpreted to be vague symptoms (Table 3). Only the GP’s symptom interpretation remained statistically significant associated with CPP referral, across cancer sites, after adjustments, except for malignant melanoma for which no association was found (Table 3)*.* Furthermore, even though no overall association between age and CPP referral was observed, breast cancer patients aged 45–64 were less likely to be referred to a CPP (Table 3).

**Table 3:** **Patient’s chance of CPP referral initiated by the GP, expressed as adjusted prevalence ratios (PRs) by cancer site and total (N=3,672)**

	Colorectal	Lung	Malignant melanoma	Breast	Prostate	Other	Total
	PRR (95% CI)	PRR (95% CI)	PRR (95% CI)	PRR (95% CI)	PRR (95% CI)	PRR (95% CI)	PRR (95% CI)
**Gender**							
Male	1.08(0.88-1.32)	1.02(0.81-1.27)	1.02(0.72-1.44)	n/a	1.00	**1.22** **(1.03-1.44)**	**1.12** **(1.00-1.24)**
Female	1.00(ref)	1.00(ref)	1.00(ref)	1.00	n/a	1.00(ref)	1.00(ref)
**Age groups (years):**							
18-44	1.67(0.87-3.18)	0.64(0.09-4.32)	1.16(0.60-2.21)	0.86(0.69-1.09)	**-**	0.98(0.71-1.34)	1.04(0.88-1.24)
45-54	1.02(0.63-1.66)	0.72(0.43-1.22)	1.09(0.54-2.19)	**0.78** **(0.64-0.96)**	1.70(0.98-2.93)	1.04(0.78-1.39)	0.97(0.84-1.12)
55-64	**1.49(1.11-2.00)**	0.94(0.68-1.29)	1.69(0.88-3.26)	**0.80** **(0.65-1.00)**	1.33(0.96-1.83)	1.05(0.82-1.36)	1.11(0.98-1.26)
65-74	1.18(0.91-1.54)	1.09(0.83-1.42)	1.39(0.72-2.68)	0.88(0.72-1.07)	**1.38** **(1.04-1.84)**	0.92(0.72-1.17)	1.06(0.95-1.18)
> = 75	1.00(ref)	1.00(ref)	1.00(ref)	1.00(ref)	1.00(ref)	1.00(ref)	1(ref)
**GP’s symptom interpretation**							
Alarm	1.00(ref)	1.00(ref)	1.00(ref)	1.00(ref)	1.00(ref)	1.00(ref)	1.00(ref)
Serious	**0.27(0.17-0.41)**	**0.64** **(0.49-0.85)**	n/a	**0.23(0.09-0.56)**	0.71(0.47-1.06)	**0.34** **(0.26-0.45)**	**0.40** **(0.34-0.48)**
Vague	**0.40(0.30-0.54)**	0.83(0.65-1.05)	0.76(0.52-1.13)	**0.27** **(0.17-0.43)**	**0.72** **(0.58-0.90)**	**0.44** **(0.35-0.55)**	**0.53** **(0.48-0.60)**

Adjusted for the patient’s gender, age, co-morbidity, educational background and disposable income, cancer site and patient clusters at GP level.

Estimates marked in bold were statistically significant at minimum level of p < 0.05.

n/a = not applicable.

### Diagnostic interval

The overall median diagnostic interval was 32 days (interquartile interval (IQI): 14–73) and varied from a median of 18 (IQI: 8–34) days for breast cancer patients to a median of 46 (IQI: 21–110) days for prostate cancer patients (p < 0.001). The diagnostic interval differed statistically significantly between GP symptom interpretation (p < 0.001) and GP referral modes (p < 0.001) (Table 4).

**Table 4:** **Unadjusted median diagnostic intervals (DIs) with inter-quartile intervals (IQI) displayed for five high incidence cancer sites and totally (N=3,823)**

	Colorectal	Lung	Malignant melanoma	Breast	Prostate	Other	Total
	Median (IQI)	Median (IQI)	Median (IQI)	Median (IQI)	Median (IQI)	Median (IQI)	Median (IQI)
**Total**	31(14;69)	28(11;67)	28(12;55)	18(8;34)	46(21;110)	40(16;88)	32(14;73)
**Gender**							
Male	**28**(13;64)	27(9;67)	22(9;53)	n/a	46(21;110)	38(15;84)	**35**(14;81)
Female	**35**(16;73)	28(13;69)	30(15;56)	18(8;34)	n/a	43(17;90)	**29**(12;63)
**Age groups (years):**							
18-44	44(31;89)	**68**(13;96)	31(11;55)	19(11;30)	n/a	45(15;116)	**30**(13;72)
45-54	31(16;61)	**18**(11;35)	23(12;45)	22(8;36)	47(25;160)	35(16;73)	**27**(13;54)
55-64	28(11;67)	**25**(8;44)	24(11;45)	15(6;32)	36(22;105)	38(15;77)	**30**(13;64)
65-74	30(14;61)	**27**(9;77)	30(15;56)	21(9;36)	46(22;97)	41(17;90)	**35**(15;78)
> = 75	34(15;83)	**33**(14;87)	36(14;68)	14(7;30)	53(17;165)	42(14;94)	**34**(13;84)
**GP’s symptom Interpretation**							
Alarm	**21**(8;41)	**15**(7;36)	**16**(6;35)	**15**(7;28)	**36**(15;84)	**25**(10;51)	**21**(8;42)
Serious	**31**(16;65)	**27**(9;65)	n/a	**29**(12;55)	**42**(25;88)	**35**(15;74)	**33**(14;72)
Vague	**61**(30;142)	**44**(21;89)	**39**(23;78)	**44**(24;66)	**59**(24;177)	**75**(38;152)	**60**(28;127)
**Referral mode**							
Cancer Patient Pathway (CPP)	**22**(8;46)	**20**(9;46)	**15**(6;29)	**13**(5;23)	**34**(19;75)	**29**(11;56)	**22**(8;44)
Cancer obs – no CPP	**29**(14;67)	**29**(12;65)	**26**(12;45)	**28**(13;43)	**43**(21;154)	**40**(15;79)	**34**(14;75)
Other	**42**(21;85)	**37**(14;89)	**56**(36;87)	**32**(18;54)	**67**(29;165)	**51**(22;116)	**49**(21;99)
Unknown	**34**(10;75)	**29**(7;69)	**20**(8;45)	**39**(17;77)	**47**(13;505)	**36**(13;98)	**33**(12;93)

Estimates marked in bold were statistically significant at minimum level of p < 0.05.

n/a = not applicable.

The adjusted diagnostic interval was longer when the GP did not suspect cancer and also when the GP did not refer to a CPP. Symptoms interpreted as ‘Vague’ displayed the strongest association with the diagnostic interval, ranging from an additional 17 (95% CI: 13;21) days at the 25th percentile to an additional 192 (95% CI: -98;483) days at the 90th percentile compared to patients, who had symptoms interpreted to be alarm symptoms (Table 5). The additional diagnostic interval that was associated with GP’s interpretation of symptoms as ‘vague’ was approximately twice as long as the additional diagnostic interval that was associated with non-CPP referral (Table 5).

**Table 5:** **Diagnostic interval in calendar days displayed by GP’s symptom interpretation, referral mode, gender, age groups and co-morbidity (N=3,672)**

		Quantile regression results (adjusted) ^1^			
		25th percentile	50th percentile	75th percentile	90th percentile
	n (%)	estimate (95% CI)	estimate (95% CI)	estimate (95% CI)	estimate (95% CI)
**Gender**					
Male	1,961 (53.4)	**-4**(-6;-1)	-1(-6;3)	-6(-21;10)	-19(-242;203)
Female	1,711 (46.6)	ref.	ref.	ref.	ref.
**Age groups (years):**					
18-44	289 (7.9)	-1(-7;4)	-1(-9;4)	-8(-22;7)	-52(-139;35)
45-54	447 (12.2)	-2(-6;2)	-6(-12;0)	-10(-25;5)	-37(-276;303)
55-64	835 (22.7)	**-**3(-6;0)	-5(-10;1)	-11(-29;7)	**-**56(-132;21)
65-74	1,129 (30.7)	0(-2;2)	0(-6;6)	-2(-21;17)	-30(-107;48)
> = 75	972 (26.5)	ref.	ref.	ref.	ref.
**GP’s symptom Interpretation**					
Alarm	1,766 (48.1)	ref.	ref.	ref.	ref.
Serious	706 (19.2)	**3**(0;6)	**8**(3;13)	**24**(7;41)	68(-102;237)
Vague	1,200 (32.7)	**17**(13;21)	**34**(28;41)	**72**(38;107)	192(-98;483)
**Referral Mode**					
CPP	1,371 (37.3)	ref.	ref.	ref.	ref.
Cancer obs. – no CPP	888 (24.2)	**5**(1;9)	**13**(7;19)	**32**(17;46)	108(-31;248)
Other reasons	1,253 (34.1)	**8**(4;11)	**16**(11;22)	**34**(17;52)	123(86;331)
Unknown	160 (4.4)	3(-7;13)	10(-10;30)	**30**(1;59)	179(-412;770)
**Co-morbidity**					
None	2,780 (75.7)	ref.	ref.	ref.	ref.
Moderate	741 (20.2)	-1(-3;1)	2(-2;7)	8(-6;23)	5(-57;66)
High	151 (4.1)	-2(-7;4)	1(-9;13)	-1(-30;28)	-27(-110;55)

Point estimates marked in bold are statistically significant at minimum level of p < 0.05.

^1^Adjusted for gender, age groups, symptom interpretation, referral mode, cancer site, comorbidity, educational background, disposable income and region of residence.

## Discussion

The GPs suspected cancer in 48.2% of all cancer patients and initiated CPP in 37.2% of all cases. Patients had a lower likelihood to be referred to a CPP if the GP interpreted symptoms as ‘vague’ or ‘serious’ compared to ‘alarm’ symptoms. Thus, the GP’s symptom interpretation increased the diagnostic interval for the group interpreted to have ‘vague’ symptoms (32.7% of all cases) and the group interpreted to have ‘serious’ symptoms (19.2% of all cases).

The GP’s symptom interpretation influenced the diagnostic interval twice as much as the referral mode chosen by the GP. This indicates that the GP-assessed severity of symptoms influences the diagnostic interval more than the GP’s choice of referral mode.

### Strengths and weaknesses of the study

The size of this study is a major strength as the considerable data ensure high statistical precision. Furthermore, the study population was well-defined and complete with minimal selection bias as all cases were identified through the NPR [23, 33], wherein 98% of all cancer patients in Denmark are registered [25]. Yet, we may have missed some patients due to delay in NPR registrations. However, this is expected to be negligible as we performed consecutive sampling (including late-registered patients) [23].

The high response rate of 74% further reduces the risk of selection bias. The small differences in gender for patients listed with responding and non-responding GPs should not affect the representativeness of the study as the cohort resembles patients in the Danish Cancer Registry [23]. However, patients who were excluded due to GP non-response may have had longer diagnostic intervals than the included patients. However, this will give minimal bias (if any) as we looked at associations between diagnostic interval and symptom presentation.

Information bias caused by GP recall bias was reduced as we used the GPs’ contemporaneously updated electronic medical records. Even so, the retrospective nature of the questionnaire may imply the risk that some of the GPs may have misinterpreted the symptoms of a particular case and hence may have overestimated the proportion of cases with ‘alarm’ symptoms. This would tend to underestimate the association between the GP’s assessment of ‘alarm-symptoms’, use of CPPs and the diagnostic interval. Yet, we believe that this cannot fully explain the proportion of patients with ‘alarm’ symptoms found in our study as other studies have found similar proportions [19, 20].

Information bias due to use of ‘date of first contact’ as ‘date of diagnosis’ would tend to underestimate the length of the diagnostic interval by setting an earlier date of diagnosis. We consider this to be non-differential as this is suspected to be the case for all subgroups and hence will not depend on the GPs symptom interpretation and choice of referral route. Yet, it could be argued that this information bias would be stronger for patients who were not referred to a CPP as these have longer intervals (and thus may have a relatively higher impact on non-CPP patients). If this is the case, this could lead to an underestimation of the differences between referral groups, and the observed differences would thus represent minimum estimates of the true differences.

### Comparison with other findings

Our finding that nearly 50% of cancer patients, who had symptoms interpreted to be ‘alarm’ symptoms of cancer prior to a cancer diagnosis represent a slightly higher number than the previously reported 40% [19, 20]. Yet, this suggests that half of all cancer patients present without an ‘alarm’ symptom of cancer. In combination with the fact that most symptoms of cancer are highly prevalent in general practice [16, 19, 20], this indicates that a patient may have cancer even if no specific alarm symptoms are presented by the patient.

Our study is the first to document the use of CPP in primary care for all cancers in Denmark. Our finding that approximately one-third of all patients are referred to a CPP is comparable to the findings on the use of 2WW in the UK [8–10, 13, 39]. The reasons for these results remain unknown, but it may be suspected that the criteria behind the ‘reasonable suspicion of cancer’, is too specific to target the patients’ symptomatology in general practice, as up to 60% of cancer patients do not present with alarm symptoms [19, 20]. This issue has also been raised as a concern in the UK [8, 13].

To our knowledge, only one study has estimated adjusted associations with the diagnostic interval at different percentiles, but this study did not adjust for cancer suspicion nor for the case-mix [40]. Hence, our study is the first to quantify the associations between cancer suspicion and diagnostic interval at different percentiles while also accounting for the case-mix. Even so, our finding of an overall (unadjusted) median diagnostic interval of one month is similar to the findings of other studies [8, 11, 12, 40–42].

The low use of CPP referrals in combination with a longer diagnostic interval for patients, whose symptoms was not interpreted as ‘alarm’ symptoms make us question if the CPP (and 2WW) approach to faster diagnosis is the optimal method to use at the starting point of the diagnostic trajectory. In fact, we have shown that lack of cancer suspicion by the GP decreases the likelihood of CPP referral and influences the diagnostic interval considerably more than the actual use of CPP, in particular among patients with vague symptoms.

We have also shown that the severity of presented symptoms was not directly associated with the GP’s use of a fast-track system. In combination with the English data that a ‘fast-track’ system may disadvantage the large group of patients without a warning sign of cancer [10, 13], our finding may be interpreted as a demonstration of the possible fallacies of the CPP and 2WW referral routes for cancer and why an additional approach with quick and easy access to all initial investigations ordered by a GP to qualify the possibility of cancer may be needed, but further research into the organisation of rational investigations is highly needed.

### Clinical implications

This study underlines the importance for clinicians in general practice to consider and investigate for cancer even when the patient does not present well-known alarm symptoms of cancer. Otherwise, only a proportion of cancer patients will be provided the faster diagnostic pathway, leaving approximately half of all cancer patients to a longer period of uncertainty before diagnosis is confirmed. This implies that the GPs must have access to relevant investigations if the aim is to achieve earlier cancer diagnosis.

## Conclusions

GPs suspected cancer more often than they initiated a CPP, and patients were less likely to be referred to a CPP if their symptoms were not interpreted to be an ‘alarm’ symptom of cancer. Furthermore, when the patient’s symptoms were interpreted by the GP as ‘vague’ , this gave rise to a significantly prolonged diagnostic interval; the impact of the symptom interpretation was approximately twice that of not using CPP referral routes. To decrease the time from first symptom presentation until diagnosis for those without alarm symptoms, GPs may need additional routes other than the fast-track routes.

## Abbreviations

CPP: Standardised Cancer Patient Pathways
GP: General practitioner
PR: Prevalence Ratio
2WW: 2 Week Wait
NPR: Danish National Patient Registry
DCR: Danish Cancer Register
CRN: Danish Civil Registration Number
CCI: Charlson Co-morbidty Index
ISCED: International Standard Classification of Education.
